# Oral Delivery of *Bacillus subtilis* Expressing Chicken NK-2 Peptide Protects Against *Eimeria acervulina* Infection in Broiler Chickens

**DOI:** 10.3389/fvets.2021.684818

**Published:** 2021-06-04

**Authors:** Samiru S. Wickramasuriya, Inkyung Park, Youngsub Lee, Woo H. Kim, Chris Przybyszewski, Cyril G. Gay, Jolieke G. van Oosterwijk, Hyun S. Lillehoj

**Affiliations:** ^1^Animal Bioscience and Biotechnology Laboratory, Agricultural Research Service, United States Department of Agriculture, Beltsville, MD, United States; ^2^College of Veterinary Medicine, Institute of Animal Medicine, Gyeongsang National University, Jinju, South Korea; ^3^US Biologic, Inc., Memphis, TN, United States; ^4^National Program Staff-Animal Health, Agricultural Research Service, United States Department of Agriculture, Beltsville, MD, United States

**Keywords:** NK-lysin, chicken, *Bacillus subtilis*, coccidiosis, antimicrobial peptide, oxidative stress, growth performance, gut health

## Abstract

Chicken NK-lysin peptide 2 (cNK-2) is a natural lytic peptide with direct cytotoxicity against many apicomplexan parasites including *Eimeria*. Developing an effective oral delivery strategy to express cNK-2 in the intestine, where *Eimeria* parasites interact with the host's gut epithelial cells, may effectively reduce the fecundity of parasites and minimize intestinal damage. Furthermore, cNK-2 modulates gut immune responses to decrease local inflammation elicited by *Eimeria* infection in the intestine. Therefore, we developed a stable strain of *Bacillus subtilis* (*B. subtilis*) that carries cNK-2 to the gut to determine its effectiveness in ameliorating the negative impacts of coccidiosis and to replace the use of antibiotics in controlling coccidiosis in commercial broiler chicken production. Chickens were randomly allocated into eight treatment groups: two control groups (NC: *E. acervulina* infected non-*B. subtilis* control; CON: non-infected control); three *B. subtilis-*empty vector (EV) groups (EV6: 10^6^ cfu/day/bird; EV8: 10^8^ cfu/day/bird; EV10: 10^10^ cfu/day/bird), and three *B. subtilis-*cNK-2 groups (NK6: 10^6^ cfu/day/bird; NK8: 10^8^ cfu/day/bird; NK10: 10^10^ cfu/day/bird). All chickens, except those in the CON group, were challenged with 5,000 freshly sporulated *E. acervulina* oocysts through oral gavage on day 15. Chickens were given an oral dose of *B. subtilis* on days 14, 15, and 16. Body weight, weight gains, and fecal oocyst shedding were measured. To investigate the efficacy of oral *B. subtilis*-cNK-2 against coccidiosis, gene expression of gut health-related biomarkers was measured using RT-PCR. Markers included SOD1, CAT, and HMOX1 for oxidative stress in the spleen and intestinal mucosa, OCLN, ZO-1, and JAM2 for tight junction proteins, and MUC2 for mucin gene expression in the gut. The results showed that oral treatment of young chickens with *B. subtilis-*cNK-2 improved growth performance, enhanced gut integrity, and reduced fecal oocyst shedding. Altogether, these results confirm *B. subtilis-*cNK-2 treatment as a promising and effective alternative strategy to replace antibiotics against coccidiosis based on its ability to reduce parasite survival, to reduce coccidiosis-induced body weight loss, and to decrease gut damage based on the enhanced expression of proteins associated with gut integrity and intestinal health.

## Introduction

Coccidiosis is a major enteric disease of chickens that is caused by several distinct species of *Eimeria* protozoan parasites infecting different areas of the gut. Coccidiosis primarily damages epithelial integrity in the intestine decreasing nutrient utilization and resulting in an annual loss of over $3.2 billion in the poultry industry globally (1–3). With the onset of coccidiosis, *Eimeria* elicits a local inflammatory response in the intestine, increasing gut permeability, and poor nutrient absorption that hinders optimal growth performance (2, 4). A live attenuated coccidiosis vaccine is a cost-effective approach currently utilized to control coccidiosis in commercial poultry. However, the inability to induce consistent protection, evidence of residual virulence, increasing incidence of drug resistance in *Eimeria*, and strict governmental regulation of in-feed medication in poultry production prompt the discovery of alternative strategies to control coccidiosis to support “no antibiotics ever” or “antibiotic-free” meat production for the poultry industry (5).

Alternative strategies to antibiotics, including recombinant vaccines, hyperimmune IgY antibodies, probiotics, prebiotics, phytochemicals, and antimicrobial peptides (AMPs) have been shown to reduce the clinical symptoms of poultry diseases, to enhance host innate immunity, and growth performance at various levels of efficacy (6–8). These alternatives have also been shown to control coccidiosis in commercial poultry by maintaining gut health and enhancing immunity. Among these alternatives, AMPs are small peptides that may have direct cytotoxic effects against various pathogens, including parasites such as *Eimeria* (1, 9). The amphipathic structure of AMP molecules easily destroys a wide range of pathogens by interacting with negatively charged cationic residues (7, 9, 10).

NK-lysin is a cationic amphiphilic AMP originally identified in Natural killer (NK) cells of porcine intestinal tissue and later characterized in chicken (1, 11). NK-lysin has a globular structure with 78 amino acid residues and is orthologous to human granulysin. NK-lysin, like other members of the saposin-like protein family, exhibits cytolytic activities against tumor cells and microbes (12). NK-lysin also mediates immune regulation at low doses, which may play a pivotal role in the induction of adaptive immunity and regulation of inflammatory response (9).

Chicken NK-lysin (cNK-2) is a natural lytic peptide that has been reported to have effective cytotoxicity against apicomplexan parasites such as *Eimeria* by disrupting the sporozoite membrane (9, 12). Chicken NK-2 derived from the cationic core region of the NK-lysin protein that is secreted from chicken cytotoxic lymphocytes during coccidiosis (10, 12, 13). The characterization and expression of cNK-2 have been previously studied and well-documented (10, 13). Additionally, cNK-2 has been shown to successfully destroy *Eimeria spp*. in both *in vitro* and *in vivo* studies (1).

Even though the structure and function of cNK-2 have been studied extensively, a major gap remains the discovery of an effective oral delivery strategy to preserve the functional activity of cNK-2 in the chicken gut under commercial operations. Without an effective delivery system, AMPs may lose their activity with the presence of enzyme degradation in the gut (9). In a sustainable delivery system, AMPs not only promote host local immunity but also reduce parasite growth and promote a healthy gut microbial community. Moreover, an effective industry-friendly delivery strategy for antibiotic alternatives will reduce the cost of labor and increase the effectiveness of antibiotic-free animal production.

Gram-positive *B. subtilis* is generally recognized as a highly resistant microbe to environmental stresses, stable, and safe environmental bacterium uniquely qualified for oral delivery of peptides (14, 15). *B. subtilis* can transit through the digestive tract, and the self-assembly structure of *Bacillus* spores demonstrate its superior ability to work as a successful delivery vehicle (16). In previous studies, recombinant *B. subtilis* spores with different protein expression levels were studied as a promising approach for sustainable delivery systems for mice, swine, and aquaculture (14, 17, 18).

To our knowledge, this is the first time *B. subtilis* has been explored for cNK-2 peptide delivery to the gut in chickens as a prevention strategy against coccidiosis. Therefore, we developed a stable strain of probiotic *B. subtilis* expressing cNK-2 to investigate its effectiveness as a carrier of cNK-2 to the gut and to explore its protective effect against coccidiosis challenge infection in commercial broiler chickens. In addition to evaluating the impact on growth and clinical signs caused by coccidiosis, we also evaluated gut integrity and immunity by looking at the presence of tight junction transcripts and antioxidative responses.

## Materials and Methods

### Recombinant *B. subtilis* Construction

Recombinant *B. subtilis* spores expressing empty vector (*B. subtilis*-EV) or *B. subtilis*-cNK-2 were constructed and provided by US Biologic (Memphis, TN). The NK-lysin used for the expression in a bacterial vector was based on the chicken NK-lysin sequence (RRQRSICKQLLKKLRQQLSDALQNNDD) reported previously (1, 9), which was then cloned into the pTTB2 expression vector (MoBiTec). Briefly, pTTB2-cNK was then expanded in BL21 competent *E. coli* (New England Biolabs, Inc., Ipswich, MA) and purified using the GeneJET Plasmid Miniprep Kit (Thermo Fischer Scientific, Madison, WI). Sequences were confirmed using Sanger sequencing and purified plasmids were religated using the Rapid DNA Ligation (Thermo Fisher Scientific, Madison, WI). Competent *B. subtilis* cells (strain WB800N, MoBiTec) were transformed using 0.1 M EGTA and expanded on agar plates using 2% xylose as a selection agent. Single colonies were sequenced and expanded using 2xYT media (Difco, BD Diagnostic Systems, Sparks, MD).

### *In vitro* Killing Assays

*B. subtilis*-EV and *B. subtilis*-cNK-2 were grown in media and culture supernatants tested for anti-sporozoite activity using an *in vitro* assay as described (19). Briefly, sporocysts from freshly sporulated *E. acervulina* oocysts were harvested and purified using isopycnic centrifugation on a Percoll gradient followed by washing with ice-cold phosphate-buffered saline. Next, sporocysts were treated with excystation solution (0.25% trypsin, 0.014 M taurocholic acid) and incubated for 30 min at 41°C to release sporozoites. Afterward, sporozoites were harvested by filtering the excystation solution and washed with Hank's balanced salt solution (HBSS; Sigma-Aldrich, St. Louis, MO, USA). *E. acervulina* sporozoites (1.0 × 10^7^/mL) were mixed with the culture supernatant from *B. subtilis*-EV or *B. subtilis*-cNK-2 culture in a 1:1 ratio. Recombinant cNK-2 (Genscript, Piscataway, NJ) was used as control at a concentration of 100 μg/mL. After 3 h incubation at 41°C, the sporozoites were stained with fluorescence viability dye (AO/PI staining solution, Nexcelom Bioscience LLC, Lawrence, MA), and viable sporozoites were counted microscopically.

### Chickens and Animal Care

Eighty one-day-old Ross broiler chicks (Ross 708) were obtained from a local hatchery (Longnecker Hatchery, Elizabethtown, PA) and housed in Petersime brooder units maintained in a temperature-controlled closed-house environment. Chickens were raised to 14 days of age with non-medicated commercial starter diets. After 14 days, chickens were moved to experimental grower cages and fed a non-medicated commercial grower diet until the end of the experimental period. *Ad libitum* feeds and fresh clean water were provided at all times.

### Experimental Design

**Figure 1:**
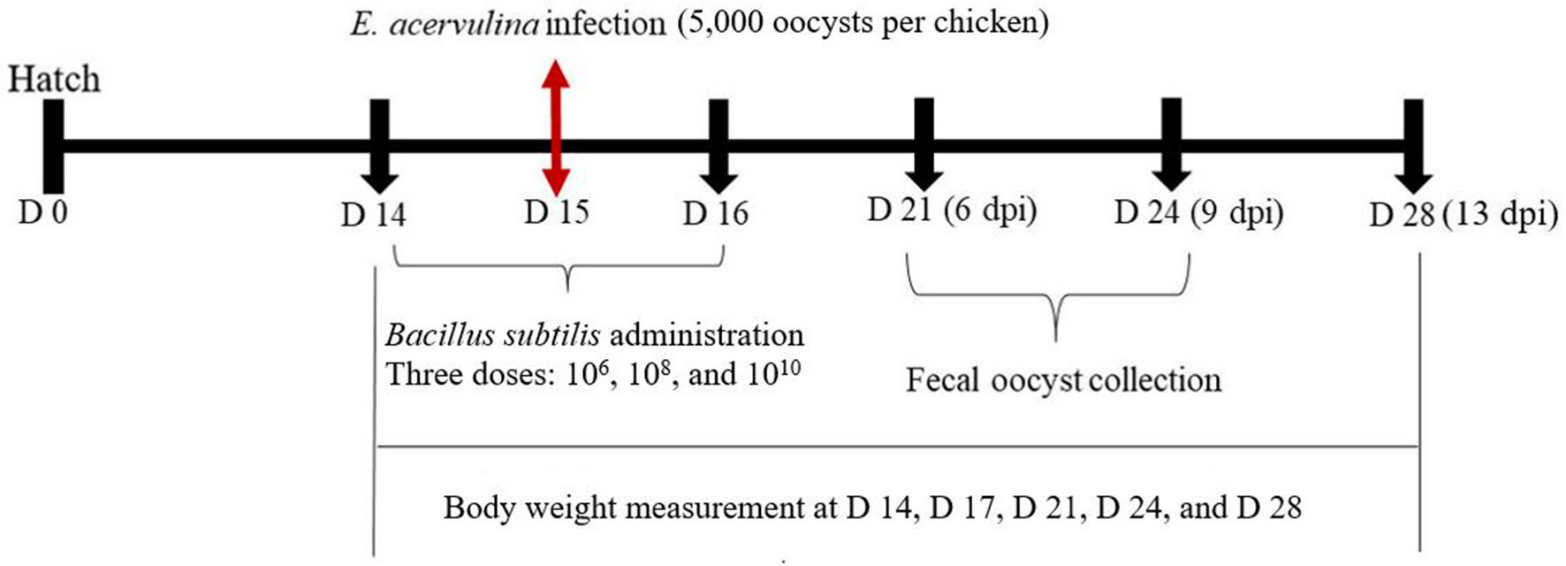
Schematic outline of the experimental design.

On day 14, body weights were recorded and chickens were randomly allocated to eight treatments (10 birds/two cages/treatment; each bird considered as a replicate), whilst ensuring similar body weight distributions among treatments and replicates. Experimental treatments included: non-infected control (CON), infected control without any *B. subtilis* (NC), the infected treatment administered with *B. subtilis-*EV at three different dosages (10^6^, 10^8^, and 10^10^ cfu/day/bird; EV6, EV8, and EV10, respectively), and the infected treatment with *B. subtilis-*cNK-2 at three different dosages (10^6^, 10^8^, and 10^10^ cfu/day/bird; NK6, NK8, and NK10, respectively) (Table 1). On days 14, 15, and 16, groups receiving *B. subtilis* were administered their dedicated dose (1 mL/bird) using oral gavage (Figure 1). Groups receiving *E. acervulina* challenge were challenged on day 15 with 5,000 freshly propagated *E. acervulina* oocysts (ARS Beltsville strain #12) (20).

**Table 1:** Treatment and *Eimeria* challenge infection.

**Treatment**	**Abbreviation**	**Description**	***B. subtilis***
			**dosage**
Non-infected group	CON	–	–
Infected group	NC	*E. acervulina*	–
*B. subtilis*-EV	EV6	*E. acervulina*/*B. subtilis*	10^6^ cfu/mL
	EV8	(EV)	10^8^ cfu/mL
	EV10		10^10^ cfu/mL
*B. subtilis-*cNK-2	NK6	*E. acervulina*/*B. subtilis*	10^6^ cfu/mL
	NK8	(cNK-2)	10^8^ cfu/mL
	NK10		10^10^ cfu/mL

### Body Weight and Sample Collection

Individual body weights were recorded for all chickens on day 14, 17, 21, 24, and 28. Pooled fecal samples were collected from each cage daily from day 21 (6 dpi) until day 24 (9 dpi). At the end of the experiment (28 days; 13 dpi), five birds from each treatment group were randomly selected for intestinal sample collection. Chickens were humanely sacrificed by cervical dislocation, and the spleen and the mucosa of mid-duodenum tissue were dissected and stored in RNAlater™ (Invitrogen Corporation, Carlsbad, CA) at −20°C.

### Fecal Oocyst Assessment

The collected fecal samples were processed according to the method previously described (21). Briefly, feces collected from individual cages were ground and homogenized with 3 L of water. Two subsamples from each cage were put into 50 mL tubes for oocyst counting. To count fecal oocysts, various dilutions were made initially to determine the optimum dilutions for enumeration of oocysts for each sample. Three different scientists independently counted oocysts microscopically using a McMaster counting chamber using a sodium chloride flotation method as described (21). The total number of oocysts shed per chicken was calculated using the following formula:

$$\begin{array}{l} \text{Total oocysts/bird = }(\text{oocyst count }\times \text{ dilution factor}\\ \times \text{ fecal sample volume/counting chamber volume})\\ \text{ / number of birds per cage}. \end{array}$$

### RNA Extraction and qRT-PCR

Collected tissue samples were gently washed with ice-cold HBSS (Sigma-Aldrich, St. Louis, MO, USA) and homogenized using a handheld homogenizer (TissueRuptor; Qiagen, Hilden, Germany). Total RNA was extracted using TRIzol reagent (Invitrogen) followed by DNase digestion as described (5). Quantification and purity were assessed using a NanoDrop spectrophotometer (NanoDrop One; Thermo Scientific) at 260/280 nm. Synthesis of cDNA was performed using a QuantiTect® Reverse Transcription Kit (Qiagen) according to the manufacturer's instructions. The gene expression levels of tight junction proteins such as junctional adhesion molecule 2 (JAM2), occluding (OCLN), and zonula occludens-1 (ZO1), mucin 2 (MUC2) expression in the duodenum samples, and antioxidant markers including superoxide dismutase 1 (SOD1), heme oxygenase 1 (HMOX1), and catalase (CAT), in both duodenum and spleen samples were investigated. All oligonucleotide primer sequences used in this experiment are shown in Table 2. The cDNA samples were diluted to 1:5 and 5-μL aliquots were used for qRT-PCR amplification. The sample was analyzed using SYBR Green qPCR Master Mix (PowerTrack, Applied Biosystems, Vilnius, Lithuania) in triplicate using Applied Biosystems QuantStudio 3 Real-Time PCR Systems (Life Technologies, Carlsbad, CA). The following PCR conditions were followed: denaturation at 95°C for 10 min followed by amplification at 60°C for 1 min for 40 cycles. Glyceraldehyde 3-phosphate dehydrogenase (GAPDH) was used as the reference gene for gene expression. For relative quantification of the gene expression levels, the logarithmic-scaled threshold cycle (Ct) values were used in the 2^−Δ*ΔCt*^ method before calculating the mean and standard error of the mean (SEM) for the references and individual targets.

**Table 2:** Quantitative real-time PCR oligonucleotide primer sequences.

**Target gene**	**Primer sequence**	**Accession No**.
GAPDH	F 5′-GGTGGTGCTAAGCGTGTTAT-3′	K01458
	R 5′-ACCTCTGTCATCTCTCCACA-3′	
JAM-2	F: 5′-AGCCTCAAATGGGATTGGATT	NM0,010,06257.1
	R: 5′-CATCAACTTGCATTCGCTTCA	
OCLN	F: 5′-GAGCCCAGACTACCAAAGCAA	NM205,128.1
	R: 5′-GCTTGATGTGGAAGAGCTTGTTG	
ZO-1	F: 5′-CCGCAGTCGTTCACGATCT	XM01,527,8981.1
	R: 5′-GGAGAATGTCTGGAATGGTCTGA	
MUC-2	F: 5′-GCCTGCCCAGGAAATCAAG	NM0,013,18434.1
	R: 5′-CGACAAGTTTGCTGGCACAT	
HMOX-1	F 5′-CTGGAGAAGGGTTGGCTTTCT-3′	NM205344
	R 5′-GAAGCTCTGCCTTTGGCTGTA-3′	
SOD1	F 5′-ATTACCGGCTTGTCTGATGG-3′	NM205064.1
	R 5′-CCTCCCTTTGCAGTCACATT-3′	
CAT	F 5′-ACTGCAAGGCGAAAGTGTTT-3′	NM001031215.1
	R 5′-GGCTATGGATGAAGGATGGA-3′	

*GAPDH, Glyceraldehyde 3-phosphate dehydrogenase; JAM-2, Junctional Adhesion Molecule 2; OCLN, Occludin; ZO-1, Zonula occludens-1; MUC-2, Mucin 2; HMOX-1, heme oxygenase 1; SOD1, Superoxide Dismutase 1; CAT, Catalase; F, forward primer; R, reverse primer*.

### Statistical Analysis

Data were analyzed using Mixed Model (PROC MIXED) in SAS (SAS Inc., Cary, NC). The individual chicken was considered the experimental unit for statistical analysis. The results are given as least-squares means and pooled SEM. *P*-values < 0.05 were considered to be significant. When the *p*-value between treatments was <0.05, homogeneous subsets were evaluated by the PDIFF option in SAS. The dose-response of growth performances were determined using the Interactive Matrix Language (IML) procedure of SAS to generate coefficients for the evenly spaced orthogonal contrasts. These coefficients generated by the IML procedure were then used in the mixed procedure for contrasts.

## Results

### *In vitro* Assay for Sporozoite Viability

**Figure 2:**
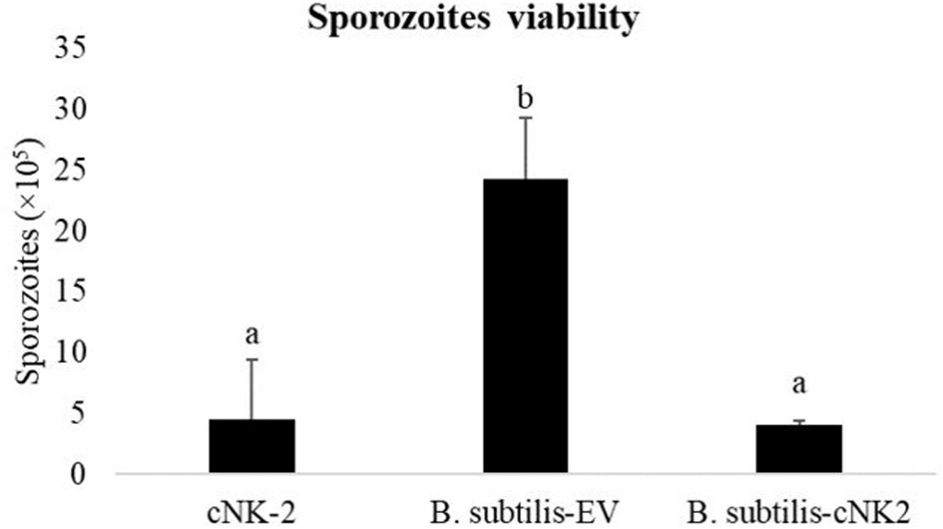
Effect of *B. subtilis* expressing empty vector (*B. subtilis*-EV) or chicken NK2 (*B. subtilis*-cNK-2) *in vitro*. *Eimeria acervulina* sporozoites (1.0 × 10^7^/mL) were incubated with culture supernatant from *B. subtilis*-EV or *B. subtilis*-cNK-2 for 3 h at 41°C. Chicken NK-lysin (cNK-2) was used as control at a concentration of 100 μg/mL. Sporozoites were stained with a fluorescence viability dye and viable sporozoites were counted microscopically. ^a, b^Bars with no common letter differ significantly (*p* < 0.05).

Sporozoites treated with cNK-2 (control) showed a significant decrease (*p* < 0.05) in sporozoites viability at the end of the 3 h incubation period (Figure 2). Culture supernatant from *B. subtilis*-cNK-2 also showed a similar (*p* > 0.05) sporozoites killing activity compared to control. However, sporozoites treated with the culture supernatant from *B. subtilis*-EV showed higher viability (*p* < 0.05) compared to control and the group treated with *B. subtilis*-cNK-2 culture.

### Body Weight and Daily Gain

Body weights of all chickens did not significantly differ (day 14: *p* > 0.05) between groups at the start of the trial (Table 3). There were no significant changes (*p* > 0.05) between treatments at day 17 (2 dpi) regardless of *E. acervulina* infection or the type and dose of *B. subtilis* administration. Chickens infected with *E. acervulina* showed lower (*p* < 0.05) body weight at day 21 (6 dpi) than the CON chickens. However, the body weights of chickens in the NK10 group were significantly higher (*p* < 0.05) than those of NC chickens at day 24 (9 dpi). Chickens that received EV did not show (*p* > 0.05) any dose responses for body weight measurement throughout the study period. In contrast, chickens in the NK groups showed enhanced body weights in a dose-dependent manner (*p* < 0.05) at day 21 and 24.

**Table 3:** Body weights of *Eimeria acervulina*-infected chickens following treatment with *Bacillus subtilis* expressing cNK-2.

										***P*****-value**		
	**CON**	**NC**	**EV6**	**EV8**	**EV10**	**NK6**	**NK8**	**NK10**	**SEM**	**Treatment**	**Linearity (dose response)**	
											**EV**	**NK**
**Body weight, g**												
D 14	397	394	398	399	390	393	392	391	14.1	1.000	0.661	0.998
D 17 (2 dpi)	579	547	566	570	591	569	565	575	15.4	0.689	0.352	0.328
D 21 (6 dpi)	854^a^	751^c^	753^c^	750^c^	749^c^	742^c^	760^c^	791^c^	20.9	0.006	0.934	0.030
D 24 (9 dpi)	1,060^a^	940^c^	944^c^	957^bc^	957^bc^	967^bc^	962^bc^	1,009^ab^	25.3	0.022	0.764	0.025
D 28 (13 dpi)	1,384^a^	1,251^b^	1,256^b^	1,263^b^	1,258^b^	1,257^b^	1,269^b^	1,301^ab^	32.3	0.079	0.996	0.154
**Average daily gain, g**												
D 14–17 (−1 to 2 dpi)	56.1	55.9	56.1	57.1	56.9	55.1	55.2	54.9	1.9	0.986	0.814	0.786
D 17–21 (2–6 dpi)	68.7^a^	51.1^bc^	46.8^cde^	45.2^cde^	39.9^e^	43.2^cde^	48.9^bc^	54.0^b^	3.5	0.001	0.013	0.011
D 21–24 (6–9 dpi)	68.8^bc^	62.5^d^	63.9^cd^	68.9^bc^	66.0^cd^	75.1^a^	67.1^bcd^	72.8^ab^	2.2	0.001	0.654	0.008
D 24–28 (9–13 dpi)	81.2	77.8	77.9	76.7	73.1	72.6	77.0	73.2	2.1	0.064	0.104	0.136
D 17–24 (2–9 dpi)	68.7^a^	56.0^c^	54.1^cd^	55.3^cd^	51.0^d^	57.0^bc^	56.8^bcd^	62.1^b^	2.0	0.001	0.125	0.007
D 17–28 (2–13 dpi)	61.9^a^	54.1^bc^	53.1^bc^	53.3^bc^	50.0^c^	52.8^bc^	54.1^bc^	56.0^b^	1.6	0.001	0.107	0.121

*D, day; dpi, days post-infection; SEM, standard error of the mean. All chickens except CON were infected by oral gavage at day 15 with 5,000 oocysts/chicken of E. acervulina. B. subtilis were administrated by oral gavage daily from d 14 to 16. EV, B. subtilis carrying empty vector; NK, Bacillus subtilis expressing cNK-2; NC, E. acervulina infected non-B. subtilis control; EV6, B. subtilis (empty vector) at 10^6^ cfu/day; EV8, B. subtilis (empty vector) at 10^8^ cfu/day; EV10, B. subtilis (empty vector) at 10^6^ cfu/day; NK6, B. subtilis expressing cNK-2 at 10^6^ cfu/day; NK8, B. subtilis expressing cNK-2 at 10^8^ cfu/day; NK10, B. subtilis expressing cNK-2 at 10^10^ cfu/day. ^a−e^Means in the same row with different superscripts differ (p < 0.05) and the difference was revaluated by PDIFF option in SAS when P-value between treatments was <0.05*.

Similar to the body weight data, no significant changes (*p* > 0.05) were seen in the average daily gain (ADG) of chickens among the different treatment groups up to day 17 (2 dpi) (Table 3). Thereafter, chickens infected with *E. acervulina* showed significantly lower (*p* < 0.05) ADG than the CON group. However, chickens in the NK10 group showed significantly increased (*p* < 0.05) ADG compared to that of the NC group from day 17 to 24 (2–9 dpi). After day 24, the NK10 group did not show any significant difference (*p* > 0.05) in ADG compared to the NC chickens regardless of NK treatment dose.

### Fecal Oocyst Shedding

**Figure 3:**
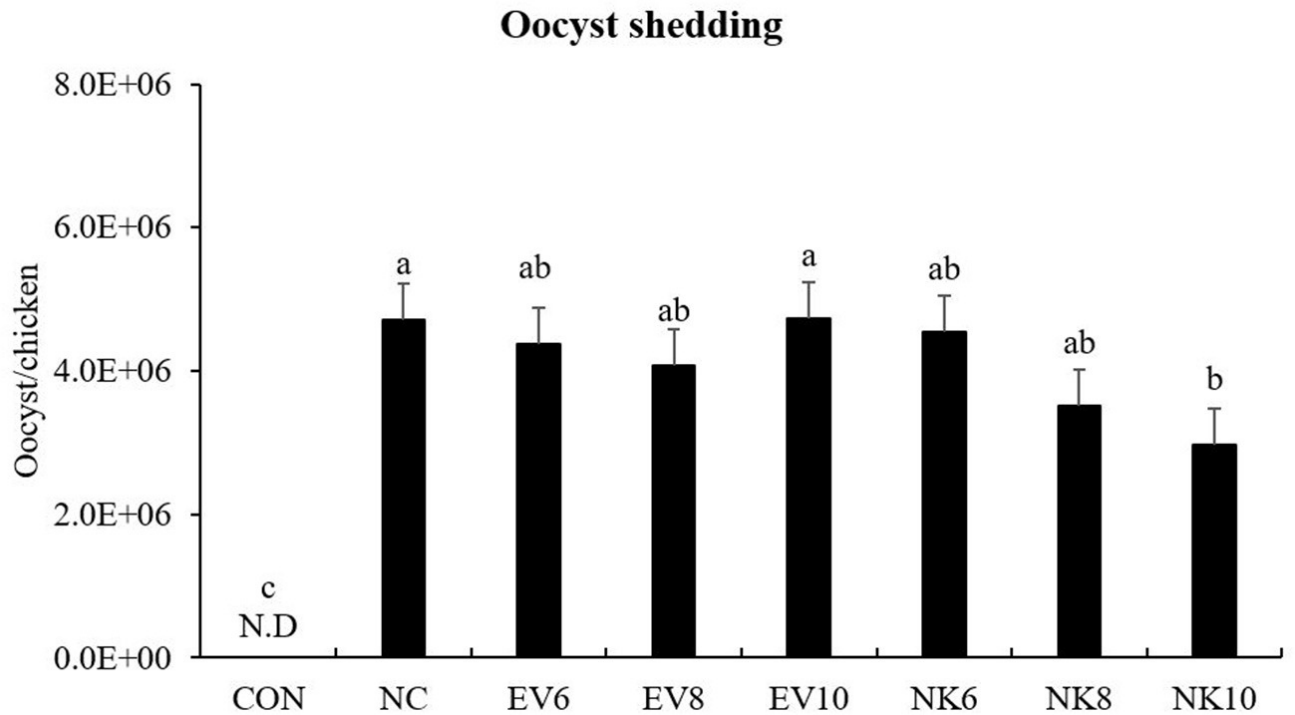
Fecal oocyst counts of *Eimeria acervulina*-infected chickens fed daily oral treatment with *Bacillus subtilis-*cNK-2. All chickens except CON were infected by oral gavage at day 15 with 5,000 oocysts/chicken of *E. acervulina*. *B. subtilis* were administrated by oral gavage at days 14–16. EV, *B. subtilis*-EV; NK, *B. subtilis-*cNK-2; NC, *E. acervulina* infected non-*B. subtilis* control; EV6, *B. subtilis* (empty vector) at 10^6^ cfu/day; EV8, *B. subtilis-*EV at 10^8^ cfu/day; EV10, *B. subtilis-*EV at 10^6^ cfu/day; NK6, *B. subtilis-*cNK-2 at 10^6^ cfu/day; NK8, *B. subtilis-*cNK-2 at 10^8^ cfu/day; NK10, *B. subtilis-*cNK-2 at 10^10^ cfu/day. ^a−*c*^Bars with no common letter differ significantly (*p* < 0.05). Each bar represents the mean ± SEM (*n* = 8). Fecal samples were collected from 6 to 9 dpi to calculate the oocyst shedding.

Chickens that were treated with an oral dose of *B. subtilis-*cNK-2 showed significantly reduced (*p* < 0.05) fecal oocyst output between 6 and 9 dpi (Figure 3). In particular, the NK10 group showed significantly decreased (*p* < 0.05) oocyst shedding compared to the NC group.

### Gene Expression of TJ Proteins and Mucin

**Figure 4:**
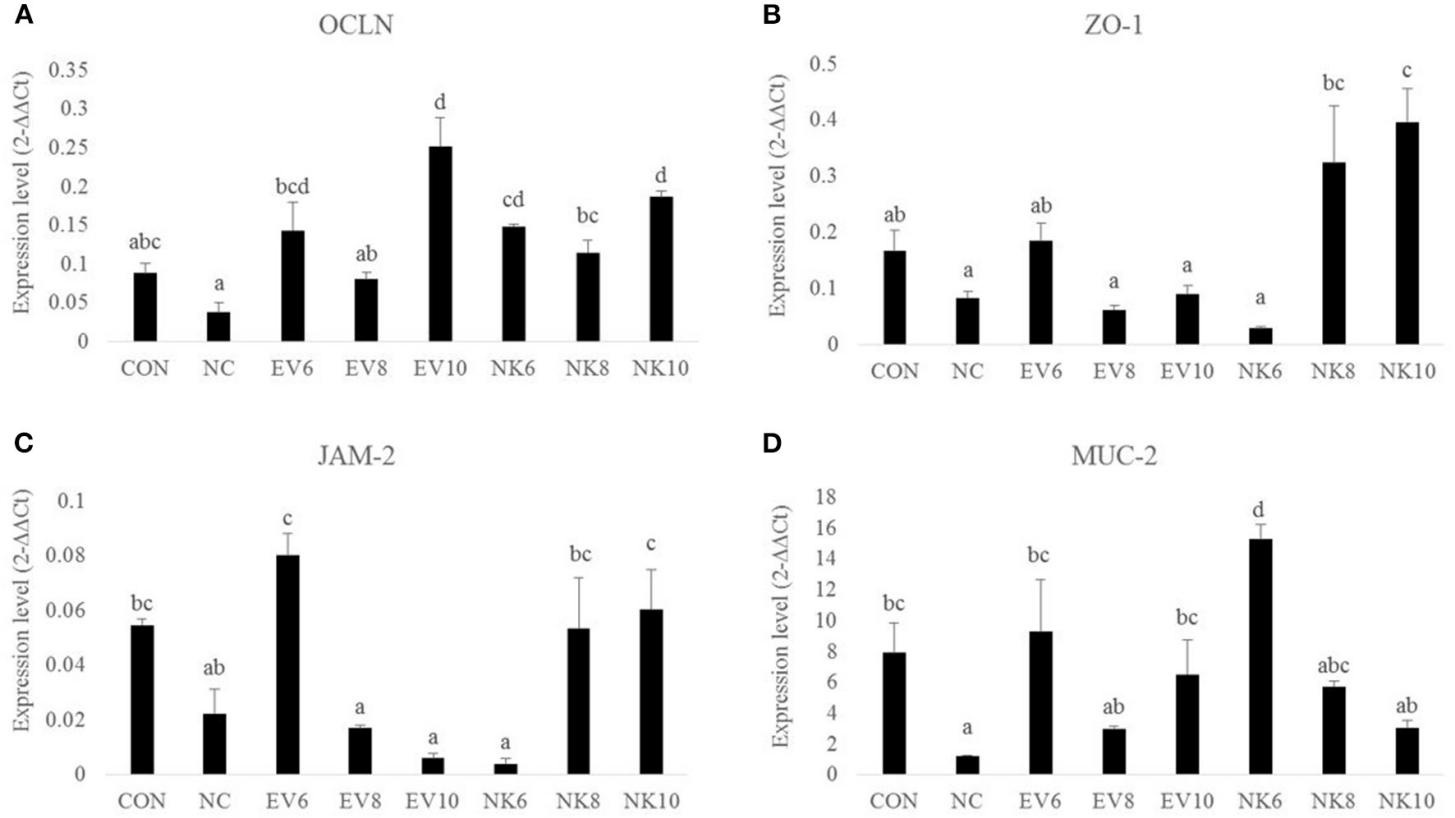
Tight junction gene expression in duodenal mucosa of *Eimeria acervulina*-infected broiler chickens fed orally *Bacillus subtilis* expressing cNK-2 (13 dpi). All chickens except CON were infected by oral gavage at day 15 with 5,000 oocysts/chicken of *E. acervulina*. *B. subtilis* was administrated by oral gavage at days 14–16. EV, *B. subtilis* (empty vector), NK, *B. subtilis-*cNK-2; NC, *E. acervulina* infected non-*B. subtilis* control; EV6, *B. subtilis-*EV at 10^6^ cfu/day; EV8, *B. subtilis-*EV at 10^8^ cfu/day; EV10, *B. subtilis-*EV at 10^10^ cfu/day; NK6, *B. subtilis-*cNK-2 at 10^6^ cfu/day; NK8, *B. subtilis-*cNK-2 at 10^8^ cfu/day; NK10, *B. subtilis-*cNK-2 at 10^10^cfu/day. Transcript levels of **(A)** occludin (OCLN), **(B)** zonula occludens-1 (ZO1), **(C)** junctional adhesion molecule 2 (JAM2), and **(D)** Mucin-2 (MUC-2) in duodenal mucosa were measured by quantitative RT-PCR and genes expression were analyzed using the 2^−Δ*ΔCt*^ method. ^a−*d*^Bars with no common letter differ significantly (*p* < 0.05). Each bar represents the mean ± SEM (*n* = 5).

Gene expression profiles of tight junction proteins in the duodenal mucosa are shown in Figure 4. On 13 dpi, chickens in the NC group did not show any significant difference (*p* > 0.05) in the expression levels of OCLN, ZO-1, and JAM-2 compared to the CON group. However, chickens that were given *B. subtilis-*cNK-2 (NK6, NK8, and NK10) showed increased (*p* < 0.05) OCLN gene expression compared to the NC chickens, regardless of doses of NK-lysin treatment. In comparison to *B. subtilis-*cNK-2 and *B. subtilis-*EV, no difference (*p* > 0.05) was observed in OCLN gene expression for each dose. Moreover, chickens in the NK8 and NK10 groups showed higher (*p* < 0.05) expression of ZO-1 than chickens in the NC, EV8, and EV10 groups. Similarly, JAM-2 expression in the duodenum was higher (*p* < 0.05) in the NK8 and NK10 treatment groups than in the EV8 and EV10 groups. Notably, MUC2 gene expression was lower (*p* < 0.05) in NC chickens than in CON chickens but increased in the EV6, EV10, and NK6 groups compared to NC chickens.

### Antioxidant Gene Expression in the Duodenal Mucosa

**Figure 5:**
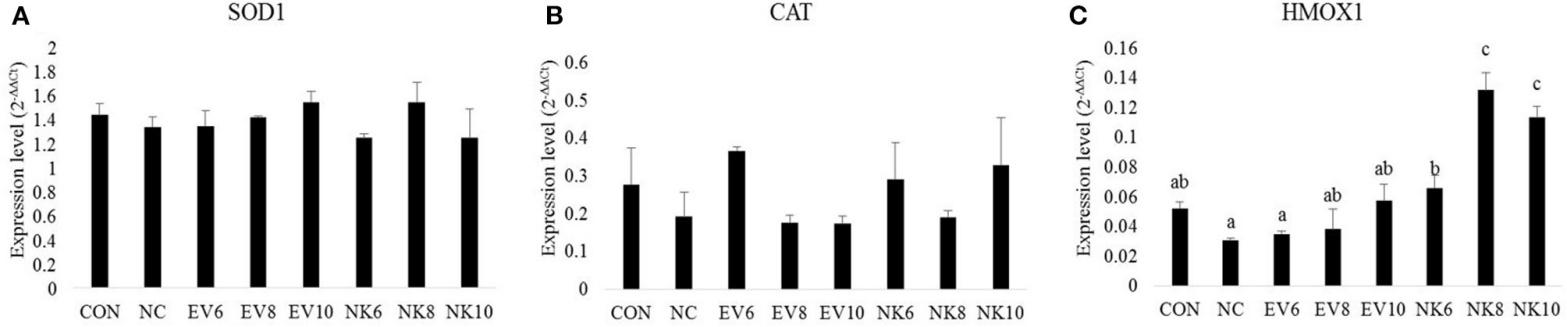
Anti-oxidant gene expression in duodenal mucosa of *Eimeria acervulina*-infected broiler chickens fed orally *Bacillus subtilis* expressing cNK-2 (13 dpi). All chickens except CON were infected by oral gavage at day 15 with 5,000 oocysts/chicken of *E. acervulina*. *B. subtilis* were administrated by oral gavage at days 14–16. EV, *B. subtilis-*EV; NK, *B. subtilis-*cNK-2; NC, *E. acervulina* infected non*-B. subtilis* control; EV6, *B. subtilis-*EV at 10^6^ cfu/day; EV8, *B. subtilis-*EV at 10^8^ cfu/day; EV10, *B. subtilis-*EV at 10^10^ cfu/day; NK6, *B. subtilis-*cNK-2 at 10^6^ cfu/day; NK8, *B. subtilis-*cNK-2 at 10^8^ cfu/day; NK10, *B. subtilis-*cNK-2 at 10^10^ cfu/day. Transcript levels of **(A)** superoxide dismutase 1 (SOD1), **(B)** catalase (CAT), **(C)** heme oxygenase (HMOX1) in duodenal mucosa were measured by quantitative RT-PCR and gene expression were analyzed using the 2^−Δ*ΔCt*^ method. ^a−*c*^Bars with no common letter differ significantly (*p* < 0.05). Each bar represents the mean ± SEM (*n* = 5).

Figure 5 shows the mucosal antioxidant gene expression profile in the duodenum. Chickens treated with *B. subtilis-*cNK-2 (NK6, NK8, and NK10) and infected with *E. acervulina* showed elevated (*p* < 0.05) expression of HMOX1 in the duodenal mucosa (Figure 5C) compared to the chickens in the *E. acervulina-*infected control (NC) group. There was no significant difference (*p* > 0.05) in the expression levels of the SOD1 and CAT genes in the duodenal mucosa at 13 dpi (Figures 5A,B).

### Antioxidant Gene Expression in Spleen

**Figure 6:**
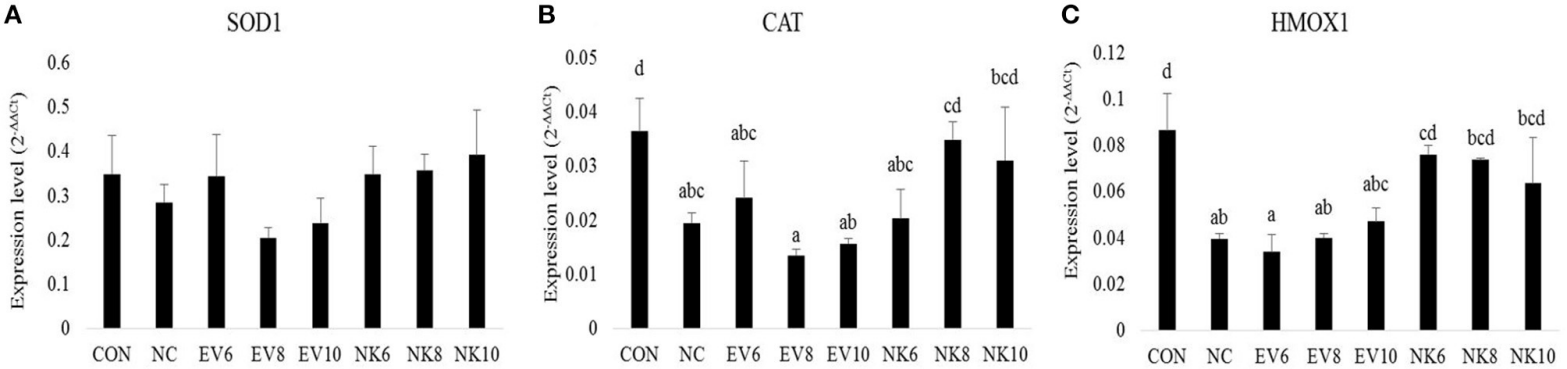
Anti-oxidant gene expression in spleen of *Eimeria acervulina*-infected broiler chickens fed orally *B. subtilis* expressing cNK-2 (13 dpi). All chickens except CON were infected by oral gavage at day 15 with 5,000 oocysts/chicken of *E. acervulina*. *B. subtilis* were administrated by oral gavage at days 14–16. EV,v*B. subtilis-*EV; NK, *B. subtilis-*cNK-2; NC, *E. acervulina* infected non-*B. subtilis* control; EV6, *B. subtilis-*EV at 10^6^ cfu/day; EV8, *B. subtilis*-EV at 10^8^ cfu/day; EV10, *B. subtilis-*EV at 10^10^ cfu/day; NK6, *B. subtilis-*cNK-2 at 10^6^ cfu/day; NK8, *B. subtilis-*cNK-2 at 10^8^ cfu/day; NK10, *B. subtilis-*cNK-2 at 10^10^ cfu/day. Transcript levels of **(A)** superoxide dismutase 1 (SOD1), **(B)** catalase (CAT), **(C)** heme oxygenase (HMOX1) in the spleen were measured by quantitative RT-PCR and genes expression were analyzed using the 2^−Δ*ΔCt*^ method. ^a−*d*^Bars with no common letter differ significantly (*p* < 0.05). Each bar represents the mean ± SEM (*n* = 5).

Gene expression profiles of antioxidant genes in the spleen are shown in Figure 6. The expression of CAT and HMOX1 was higher (*p* < 0.05) in the CON group than in the NC and EV groups. Notably, chickens orally treated with higher doses of *B. subtilis-*cNK-2 (NK8 and NK10) showed similar (*p* > 0.05) levels of expression as those of the CON group.

## Discussion

Antibiotic alternatives including host defensin proteins such as NK-lysin are becoming important feed additives for the animal industry in the post-antibiotic era (22). Antimicrobial peptides elicit first-line defense against pathogens in livestock animals and are recognized as a potential antibiotic alternative (9, 12). Among these peptides, the cNK-lysin peptide was reported to exert antibacterial activity by damaging bacterial cells (12). Nevertheless, information concerning the efficient delivery systems to deliver these AMPs to the gut are scantly documented in poultry and livestock. Hence, the development of an effective oral delivery system that could deliver AMP across the gut lumen and stimulate immune responses is needed. To this end, this study was conducted to determine the effectiveness of stable *B. subtilis* that carries cNK-2 peptide (*B. subtilis*-cNK-2) as a carrier of NK-lysin peptide to the chicken gut and to investigate its effect against *E. acervulina* infection in commercial broiler chickens. To the best of our knowledge, this study is the first to investigate and describe the potential effectiveness of oral delivery of stable *B. subtilis* as a carrier for the cNK-2 peptide against coccidiosis infection in chickens.

Diversified approaches based on nanoparticles, natural polysaccharides, bacteria, and phages have been reported as promising delivery vehicles for AMPs (23, 24). Among these AMP delivery systems, *B. subtilis* has shown unique properties as an effective carrier for AMPs in previous studies with fish, mice, and swine (14, 17, 18). Higher stability and extreme resistance to harsh environments, non-pathogenic nature, and ability to produce an industrial scale are the uniqueness of *B. subtilis* as an attractive delivery system (14, 16).

To achieve our study objective, we followed the mode of action based on the NK-lysin peptide, which displays strong cytotoxicity against *E. acervulina* as a consequence of altering the sporozoite outer membrane integrity and releasing intracellular contents (1). Controlling *E. acervulina* sporozoites invading gut epithelial cells will reduce gut damage and minimize production losses due to coccidiosis. Our *in-vitro* assay confirmed that the culture supernatant from *B. subtilis*-cNK-2 but not *B. subtilis*-EV contained anti-sporozoite activity against freshly prepared sporozoites.

In the present study, chickens in the *Eimeria*-infected control treatment showed lower growth performance than their counterpart chickens in the uninfected control, as expected. Chickens infected with *E. acervulina* showed reduced growth performance because of the interruption of normal gut physiology and nutrition metabolism (20, 25). Rochell et al. (26) and Kim et al. (27) also reported that *E. acervulina* infection reduces growth performance in broiler chickens. Our results show that *B. subtilis-*cNK-2 treatment had a significant positive effect on the growth performance of coccidia-infected broiler chickens. Importantly, there was no growth improvement in chickens given an oral dose of *B. subtilis-*EV and infected with *E. acervulina* confirming that cNK-2 delivered by *B. subtilis* is responsible for reduced parasite fecundity and minimal growth depression in response to coccidia challenge in chickens. Consistent with our findings, Lee et al. (1) reported improved body weight gain in *E. acervulina*-infected and cNK-2 peptide-treated broiler chickens. Findings from this study showing that the cNK-2 directly improved growth performance provides a strong indication that a stable *B. subtilis* delivery system is a promising way to preserve cNK-2 functionality in the chicken gut. It was also noticeable that the effect of *E. acervulina* infection on growth performance was more profound at the early stage of post-infection and was not significant in the later stages of the infection. These findings may be observed because the infection effect was reduced, and chickens began a recovery phase.

Enumeration of oocyst shedding in fecal samples is a commonly used measurement for understanding the severity of coccidiosis infections in broiler chickens. The reduction of oocysts is also used as a tool to determine the protective indices of different anti-parasite strategies against *Eimeria* invasion. In this study, *E. acervulina-*infected chickens exhibited elevated fecal oocyst shedding, confirming the establishment of *Eimeria* in the chicken gut. Chickens that were administered an oral dose of *B. subtilis*-EV showed numerically lower oocyst shedding than the infected control chickens, although no statistical significance was reached. Importantly, chickens that were given an oral dose of *B. subtilis-*cNK-2 showed a negative dose-dependent response to fecal oocyst shedding. Chickens given *B. subtilis-*cNK-2 at 10^10^ cfu/day showed significantly lower oocyst shedding at 9 dpi than untreated infected control. This outcome supports the direct effect of cNK-2 activity against *Eimeria* which confirms our previous work (1, 28). This indicates that the *B. subtilis*-cNK-2 at 10^10^ cfu/day can be used as a remedial measure to control coccidiosis in the poultry industry. By preserving cNK-2 activity against *Eimeria* parasites in the chicken gut environment, *B. subtilis* carriers are an effective and practical oral delivery vehicle for immunotherapeutic peptides such as AMP.

Tight junctions of gut epithelial cells work as a first-line defense system to protect animals from pathogen translocation by maintaining proper tight junction integrity (29). Tight junctions are comprised of a multiprotein complex, such as OCLN, ZO-1, and JAM-2, at the apical end of epithelial cells that closely contact with the lumen (4, 30). Maintaining epithelial integrity by maintaining intestinal permeability enhances the gut health of chickens and nutrient absorption to improve growth performance (30). Based on our current study, higher dosages of *B. subtilis-*cNK-2 increased tight junction gene expression in the duodenal mucosa compared to the infected control group. Recently, we showed that upregulation of these genes improved gut barrier function in the chicken intestine after *E. acervulina* infection (5). Moreover, elevated levels of ZO-1 and JAM-2 gene expression in chickens that were treated with higher dosages of *B. subtilis-*cNK-2 compared to control *B. subtilis*-EV demonstrated reduced damage by coccidiosis on gut barrier functions. Similar to tight junctions, the mucosal barrier defends against the invasion of bacteria, parasites, and other harmful species into the host (7). Mucins are glycoproteins that form a mucus barrier over the epithelial cell surfaces of the intestine (31, 32). Among mucins, MUC-2 gene expression has been identified as a gut health marker in chicken and other poultry species, as it is the major mucin produced by goblet cells (7, 31). Our results indicate that *E. acervulina-*infected chickens showed significantly decreased mucosal MUC-2 gene expression levels compared to the non-infected control. This result provides firm confirmation of mucus barrier failure in chickens challenged with coccidiosis. Chickens that were given an oral dose of *B. subtilis-*cNK-2 counteracted *E. acervulina* infection-induced MUC-2 downregulation and increased its expression. This finding confirms previous studies showing increased MUC-2 gene expression in chickens fed *B. subtilis* as a probiotic supplement (4, 7, 33).

Previously, it was reported that coccidiosis triggers oxidative stress, thereby weakening intestinal barrier functions in chickens (34). For a robust understanding of gut health improvement with *B. subtilis-*cNK-2, we also investigated mucosal oxidative stress markers. Higher production of reactive oxygen species (ROS) and reactive nitrogen species (RNS) cause imbalances between free radical production and endogenous antioxidant defense, creating oxidative stress in intestinal cells and leading to lipid peroxidation, DNA damage, and apoptosis (34). Avoiding oxidative stress, free radical formation is prevented by the inactivation of precursors of free radicals or catalysts with the aid of enzymes, such as catalase, superoxide dismutase (SOD), and glutathione peroxidase (35). SOD enzymes catalyze the conversion of superoxide ions (${\text{O}}_{2}^{-}$) to hydrogen peroxide (H_2_O_2_) and oxygen (O_2_) (4), whereas CAT enzymes catalyze the breakdown of hydrogen peroxide (H_2_O_2_) to H_2_O and O_2_ (35). HMOX1 is a rate-limiting enzyme responsible for heme-to-biliverdin catabolism (36). In this study, we did not observe any significant differences in SOD1 and CAT gene expression levels in the duodenal mucosa. However, significantly upregulated HMOX1 gene expression was observed in the duodenal mucosa from the *B. subtilis*-cNK-2-treated chickens compared to the untreated infected control. Compared to the mucosa, the spleen showed more pronounced antioxidant gene expression even though SOD1 expression remained unchanged. Both CAT and HMOX1 expression levels in the spleen were elevated with *B. subtilis-*cNK-2 treatments in this study. Although elevated expression levels of CAT and HMOX1 in the *B. subtilis-*cNK-2 treated group was not significantly different from those in the infected control group, the results support the notion that cNK-2 reduced the oxidative stress induced by *E. acervulina* infection.

## Conclusion

This study shows the effectiveness of a stable *B. subtilis* carrying the cNK-2 as an oral delivery method for NK-lysin peptide to the *E. acervulina*-infected chicken gut, which resulted in improved growth performance via enhanced gut integrity and reduced oocyst shedding. These results also help to characterize the ability of cNK-2 to reduce oxidative stress induced by *E. acervulina* infection in chickens. These findings indicate the need for future studies on the mechanisms of the *B. subtilis* carrier system for successful AMP delivery and research on the NK-lysin approach as a strategy to reduce the coccidiosis-induced gut pathology in chickens. Further research characterizing the responses of higher doses of *B. subtilis* that carry cNK-2 together with different administration intervals and mode of delivery of *Bacillus* spores using different *Eimeria* challenges is warranted to develop effective commercializable strategies to replace antibiotics.

## Data Availability Statement

The raw data supporting the conclusions of this article will be made available by the authors, without undue reservation.

## Ethics Statement

The animal study was reviewed and approved by Beltsville Agricultural Research Center Small Animal Care Committee (Animal Protocol No. 20-002).

## Author Contributions

HL, JO, IP, WK, and CG designed the research. IP, SW, WK, and YL conducted the research and analyzed data. JO produced and provided test materials for studies. SW, CG, and HL drafted the manuscript, and all authors reviewed and edited the manuscript to its final version. SW, IP, YL, WK, JO, CP, CG, and HL had responsibility for the content. All authors contributed to the article and approved the submitted version.

## Conflict of Interest

JO and CP are employed by US Biologic, Inc. The remaining authors declare that the research was conducted in the absence of any commercial or financial relationships that could be construed as a potential conflict of interest.

## References

[1] Lee SH Lillehoj HS Tuo W Murphy CA Hong YH Lillehoj EP. Parasiticidal activity of a novel synthetic peptide from the core α-helical region of NK-lysin. Vet Parasitol. (2013) 197:113–21. 10.1016/j.vetpar.2013.04.020 23664157

[2] Kim WH Chaudhari AA Lillehoj HS. Involvement of T cell immunity in avian coccidiosis. Front. Immunol. (2019) 10:2732. 10.3389/fimmu.2019.02732 31824509 PMC6886378

[3] Lu M Li RW Zhao H Yan X Lillehoj HS Sun Z. Effects of *Eimeria maxima* and *Clostridium perfringens* infections on cecal microbial composition and the possible correlation with body weight gain in broiler chickens. Res Vet Sci. (2020) 132:142–9. 10.1016/j.rvsc.2020.05.013 32575030

[4] Chaudhari AA Lee Y Lillehoj HS. Beneficial effects of dietary supplementation of *Bacillus* strains on growth performance and gut health in chickens with mixed coccidiosis infection. Vet Parasitol. (2020) 277:109009. 10.1016/j.vetpar.2019.109009 31862509

[5] Park I Lee Y Goo D Zimmerman NP Smith AH Rehberger T. The effects of dietary *Bacillus subtilis* supplementation, as an alternative to antibiotics, on growth performance, intestinal immunity, and epithelial barrier integrity in broiler chickens infected with *Eimeria maxima*. Poult Sci. (2020) 99:725–33. 10.1016/j.psj.2019.12.002 32036975 PMC7587808

[6] Lillehoj H Oh S. Phytonutrients as non-nutritive feed additives to enhance growth and host immunity in broiler chickens. J Anim Sci. (2016) 94:496. 10.2527/jam2016-1035

[7] Gadde U Kim WH Oh ST Lillehoj HS. Alternatives to antibiotics for maximizing growth performance and feed efficiency in poultry: a review. Anim Health Res Rev. (2017) 8:26–45. 10.1017/S1466252316000207 28485263

[8] Lillehoj H Liu Y Calsamiglia S Fernandez-Miyakawa ME Chi F Cravens RL. Phytochemicals as antibiotic alternatives to promote growth and enhance host health. Vet Res. (2018) 49:1–8. 10.1186/s13567-018-0562-6 30060764 PMC6066919

[9] Kim WH Lillehoj HS Min W. Evaluation of the immunomodulatory activity of the chicken NK-lysin-derived peptide cNK-2. Sci Rep. (2017) 7:1. 10.1038/srep45099 28332637 PMC5362811

[10] Hong YH Lillehoj HS Siragusa GR Bannerman DD Lillehoj EP. Antimicrobial activity of chicken NK-lysin against *Eimeria* sporozoites. Avian Dis. (2008) 52:302–5. 10.1637/8083-072307-ResNote.1 18646461

[11] Andersson M Gunne H Agerberth B Boman A Bergman T Sillard R. NK-lysin, a novel effector peptide of cytotoxic T and NK cells. Structure and cDNA cloning of the porcine form, induction by interleukin 2, antibacterial and antitumour activity. EMBO J. (1995) 14:1615–25. 10.1002/j.1460-2075.1995.tb07150.x 7737114 PMC398254

[12] Lee MO Jang HJ Han JY Womack JE. Chicken NK-lysin is an alpha-helical cationic peptide that exerts its antibacterial activity through damage of bacterial cell membranes. Poult Sci. (2014) 93:864–70. 10.3382/ps.2013-03670 24706963

[13] Hong YH Lillehoj HS Dalloul RA Min W Miska KB Tuo W. Molecular cloning and characterization of chicken NK-lysin. Vet Immunol Immunopathol. (2006) 110:339–47. 10.1016/j.vetimm.2005.11.002 16387367

[14] Ning D Leng X Li Q Xu W. Surface-displayed VP28 on *Bacillus subtilis* spores induce protection against white spot syndrome virus in crayfish by oral administration. J Appl Microbiol. (2011) 111:1327–36. 10.1111/j.1365-2672.2011.05156.x 21933311

[15] Zhang X Al-Dossary A Hussain M Setlow P Li J. Applications of *Bacillus subtilis* spores in biotechnology and advanced materials. Appl Environ. Microbiol. (2020) 18:86. 10.1128/AEM.01096-20 32631858 PMC7440806

[16] Ricca E Baccigalupi L Cangiano G De Felice M Isticato R. Mucosal vaccine delivery by non-recombinant spores of *Bacillus subtilis*. Microb Cell Fact. (2014) 13:1–9. 10.1186/s12934-014-0115-2 25112405 PMC4249717

[17] Ciabattini A Parigi R Isticato R Oggioni MR Pozzi G. Oral priming of mice by recombinant spores of *Bacillus subtilis*. Vaccine. (2004) 22:4139–43. 10.1016/j.vaccine.2004.05.001 15474704

[18] Li W Feng J Li J Li J Wang Z Khalique A. Surface display of antigen protein VP8^*^ of porcine rotavirus on *Bacillus Subtilis* spores using CotB as a fusion partner. Molecules. (2019) 24:3793. 10.3390/molecules24203793 31652492 PMC6833084

[19] Park I Goo D Nam H Wickramasuriya SS Lee K Zimmerman NP. Effects of dietary maltol on innate immunity, gut health, and growth performance of broiler chickens challenged with *Eimeria maxima*. Front Vet Sci. (2021). 10.3389/fvets.2021.667425 PMC8173067 34095279

[20] Panebra A Lillehoj HS. *Eimeria tenella* elongation factor-1α (EF-1α) coadministered with chicken IL-7 (chIL-7) DNA vaccine emulsified in Montanide Gel 01 adjuvant enhanced the immune response to *E. acervulina* infection in broiler chickens. Avian Dis. (2019) 63:342–50. 10.1637/11976-092418-Reg.1 31251536

[21] Lee YS Lee SH Gadde UD Oh ST Lee SJ Lillehoj HS. *Allium hookeri* supplementation improves intestinal immune response against necrotic enteritis in young broiler chickens. Poult Sci. (2018) 97:1899–908. 10.3382/ps/pey031 29538713

[22] Kim WH Lillehoj HS. Immunity, immunomodulation, and antibiotic alternatives to maximize the genetic potential of poultry for growth and disease response. Anim Feed Sci Technol. (2019) 250:41–50. 10.1016/j.anifeedsci.2018.09.016

[23] Callaway TC Lillehoj H Chuanchuen R Gay CG. Alternatives to antibiotics: a symposium on the challenges and solutions for animal health and production. Antibiotics. (2021) 10:471. 10.3390/antibiotics10050471 33918995 PMC8142984

[24] Mohtashamian S Boddohi S. Nanostructured polysaccharide-based carriers for antimicrobial peptide delivery. J Pharm Investig. (2017) 47:85–94. 10.1007/s40005-016-0289-1

[25] Lee SH Lillehoj HS Dalloul RA Park DW Hong YH Lin JJ. Influence of Pediococcus-based probiotic on coccidiosis in broiler chickens. Poult Sci. (2007) 86:63–6. 10.1093/ps/86.1.63 17179417

[26] Rochell SJ Parsons CM Dilger RN. Effects of *Eimeria acervulina* infection severity on growth performance, apparent ileal amino acid digestibility, and plasma concentrations of amino acids, carotenoids, and α1-acid glycoprotein in broilers. Poult Sci. (2016) 95:1573–81. 10.3382/ps/pew035 26933234

[27] Kim DK Lillehoj HS Lee SH Lillehoj EP Bravo D. Improved resistance to *Eimeria acervulina* infection in chickens due to dietary supplementation with garlic metabolites. Br J Nutr. (2013) 109:76–88. 10.1017/S0007114512000530 22717023

[28] Lillehoj HS Lee SH Hong YH. Antimicrobial Activity of Chicken NK-2 Peptide Against Apicomplexan Protozoa. United States patent US 8,691,943. Washington, DC: US Department of Agriculture (2014).

[29] Macelline SP Wickramasuriya SS Cho HM Kim E Shin TK Hong JS. Broilers fed a low protein diet supplemented with synthetic amino acids maintained growth performance and retained intestinal integrity while reducing nitrogen excretion when raised under poor sanitary conditions. Poult Sci. (2020) 99:949–58. 10.1016/j.psj.2019.10.035 32036986 PMC7587901

[30] Grant AQ Gay CG Lillehoj HS. *Bacillus* spp. as direct-fed microbial antibiotic alternatives to enhance growth, immunity, and gut health in poultry. Avian Pathol. (2018) 47:339–51. 10.1080/03079457.2018.1464117 29635926

[31] Chen J Tellez G Richards JD Escobar J. Identification of potential biomarkers for gut barrier failure in broiler chickens. Front Vet Sci. (2015) 2:14. 10.3389/fvets.2015.00014 26664943 PMC4672187

[32] Xie Z Zhao Q Wang H Wen L Li W Zhang X. Effects of antibacterial peptide combinations on growth performance, intestinal health, and immune function of broiler chickens. Poult Sci. (2020) 99:6481–92. 10.1016/j.psj.2020.08.068 33248563 PMC7810918

[33] Aliakbarpour HR Chamani M Rahimi G Sadeghi AA Qujeq D. The *Bacillus subtilis* and lactic acid bacteria probiotics influences intestinal mucin gene expression, histomorphology and growth performance in broilers. Asian Aust J Anim Sci. (2012) 25:1285. 10.5713/ajas.2012.12110 25049692 PMC4092943

[34] Mishra B Jha R. Oxidative stress in the poultry gut: potential challenges and interventions. Front Vet Sci. (2019) 6:60. 10.3389/fvets.2019.00060 30886854 PMC6409315

[35] Surai PF Kochish II Fisinin VI Kidd MT. Antioxidant defence systems and oxidative stress in poultry biology: an update. Antioxidants. (2019) 8:235. 10.3390/antiox8070235 31336672 PMC6680731

[36] Oh S Gadde UD Bravo D Lillehoj EP Lillehoj HS. Growth-promoting and antioxidant effects of *magnolia* bark extract in chickens uninfected or co-infected with *Clostridium perfringens* and *Eimeria maxima* as an experimental model of necrotic enteritis. Curr Dev Nutr. (2018) 2:nzy009. 10.1093/cdn/nzy009 30019032 PMC6041942

